# Small Intestine Microbiome and Metabolome of High and Low Residual Feed Intake Angus Heifers

**DOI:** 10.3389/fmicb.2022.862151

**Published:** 2022-04-21

**Authors:** Yue Liu, Chang Liu, Hao Wu, Qingxiang Meng, Zhenming Zhou

**Affiliations:** State Key Laboratory of Animal Nutrition, College of Animal Science and Technology, China Agricultural University, Beijing, China

**Keywords:** small intestine, microbiota, metabolomics, residual feed intake, beef cattle

## Abstract

The gastrointestinal tract (GIT) contains complex microbial communities and plays an essential role in the overall health of the host. Previous studies of beef cattle feed efficiency have primarily concentrated on the ruminal microbiota because it plays a key role in energy production and nutrient supply in the host. Although the small intestine is the important site of post-ruminal digestion and absorption of nutrients, only a few studies have explored the relationship between the microbial populations in the small intestine and feed efficiency. Moreover, variations in GIT metabolites contribute to differences in feed efficiency. The objective of this study was to investigate relationships among bacterial populations of duodenum, jejunum, ileum; microbial metabolites; and RFI phenotype of beef cattle. We carried out by using Illumina MiSeq sequencing of the 16S rRNA V3-V4 region and liquid chromatography-mass spectrometry (LC–MS). In the duodenum, the relative abundances of Firmicutes ( *p* < 0.01), *Lachnospiraceae*, *Ruminococcaceae*, *Family_XIII*, *Christensenellaceae*, *Christensenellaceae_R-7_group* ( *p* < 0.05), and *Lachnospiraceae_NK3A20_group* ( *p* < 0.05) were higher in the low residual feed intake (LRFI) group compared with the high residual feed intake (HRFI) group, whereas the HRFI group had higher abundances of Proteobacteria and *Acinetobacter* ( *p* < 0.01). In the jejunum, the relative abundances of *Lachnospiraceae* and *Lachnospiraceae_NK3A20_group* were higher in the LRFI group ( *p* < 0.05). In the ileum, the relative abundances of *Ruminococcaceae* ( *p* < 0.01), *Christensenellaceae*, *Christensenellaceae_R-7_group*, and *Ruminococcus_2* were also higher in the LRFI group ( *p* < 0.05). Moreover, the genera *Lachnospiraceae_NK3A20_group, Christensenellaceae_R-7_group*, and *Ruminococcus_2* were negatively associated with RFI, while the genus *Acinetobacter* was positively associated with RFI. The metabolomics analysis revealed that the LRFI group significantly improved protein digestion and absorption, as well as glycerophospholipid metabolism in the duodenum, jejunum, ileum. The correlation between intestinal microorganisms and metabolites revealed that some microorganisms play an important role in amino acid metabolism, glycerophospholipid metabolism, nutrient digestion and absorption, and antioxidant enhancement. The present study provides a better understanding of the small intestinal microbiota and metabolites of beef cattle with different RFI phenotypes and the relationships among them, which are potentially important for the improvement of beef cattle feed efficiency.

## Introduction

In beef cattle production, the feed cost according for 60%–75% of the total cost (Herd et al., 2003), requiring beef cattle producers to focus on the feed efficiency trait (Schnepf, 2020). Residual feed intake (RFI) is a good measure of feed efficiency, which is defined as the difference between the actual dry matter intake (DMI) and the predicted DMI based on body size and growth (Herd et al., 2003; Nkrumah et al., 2006). Additionally, RFI is a negative selection trait (Crowley et al., 2010), low-RFI cattle typically consume less feed than high-RFI cattle, which leads to the conclusion that low-RFI cattle are more profitable than high-RFI cattle; therefore, the lower RFI, the higher feed efficiency (Herd et al., 2003). The gastrointestinal tract (GIT) contains complex microbial communities that play an essential role in metabolic, physiological, immunological processes, and the overall health of the host (Fujimura et al., 2010; Hanning and Diaz-Sanchez, 2015). Therefore, studying the differences in the microbial community across the ruminant GIT might provide the relationship between the RFI phenotypes and the microbial of GIT. Because of rumen plays a key role in energy production to the ruminants, many studies of feed efficiency in beef cattle focused on the ruminal microbiota (Jewell et al., 2015; Myer et al., 2015a). The small intestine (SI) is the important site of post-ruminal digestion and absorption of nutrients in beef cattle; however, only a few studies have explored the link between the microbial populations within the lower GIT and the feed efficiency of beef cattle (Myer et al., 2015b, 2016a,b, 2017; Lopes et al., 2019). Especially, a comparison of the segments of the SI (Perea et al., 2017; Freetly et al., 2020). Additionally, microorganisms in different GIT locations might contribute separately to the RFI phenotype of beef cattle. The GIT metabolites are intermediates or products of metabolic processes, and variations in those metabolites contribute to differences in beef cattle feed efficiency (Saleem et al., 2013; Clemmons et al., 2020). It is well known that 16S rRNA sequencing technology is a mature method to analyze cattle GIT bacteria, which has been used to characterize bacterial phylogeny and taxonomy under various experimental parameters and environmental factors. Increasing numbers of studies have used 16S rRNA sequencing technology as a genetic marker to examine multiple factors associated with the cattle GIT bacteria, such as feed efficiency (Kim et al., 2017a,b; McGovern et al., 2018). Besides, metabolomics analysis produces a snapshot of the GIT environment by profiling comprehensively the metabolite abundance in biological samples (Goldansaz et al., 2017). Liquid chromatography-mass spectrometry (LC–MS) technology has been used widely to identify metabolites that differ in beef cattle with high or low feed efficiency (Clemmons et al., 2017; Artegoitia et al., 2019).

This study investigated relationships among bacterial populations of duodenum, jejunum, ileum; microbial metabolites; and the RFI phenotype of beef cattle. We hypothesized that the changes in the composition of small intestinal bacteria and metabolites are associated with the RFI phenotype of beef cattle. Moreover, we hope to provide a more comprehensive analysis of the biological determinants of SI environment and RFI phenotype in beef cattle through correlation analysis.

## Materials and Methods

### Animals, Diets, Calculation of RFI, Heifer Selection, and Sampling

The animal care and experimental procedures in this study were carried out according to the guidelines of the Laboratory Animal Welfare and Animal Experiment Ethical Committee of China Agricultural University and with their approval (Protocol No. AW08059102-2). A total of 42 Angus heifers (410 ± 25 kg live weight, aged 15 months) were fed with a diet containing 50% concentrate and 50% forage (Supplementary Table 1) according to National Academies of Sciences Engineering and Medicine (2016), and the trial lasted for 144 days (21 days of adaptation, and 123 days of data collection). During this experiment, all conditions were consistent, heifers had *ad libitum* access to water and feed. Feeding tank automatic records feed intake of each heifer’s through electronic ear tag (Zhenghong Agriculture and Animal Husbandry Machinery and Equipment Co, Shanghai, China). Weigh at the beginning, the end, as well as 14-days intervals of the experiment. The average daily gain (ADG) was computed as the coefficient of the linear regression of body weight (BW; kg) on time (d) using the PROC REG component of the SAS package (SAS Inst., Inc., Cary, NC, United States). The metabolic body weight (MBW) was computed as the midtest BW^0.75^ (Nkrumah et al., 2004). The expected DMI of each heifer was modeled and predicted by the MBW, ADG, and actual DMI with PROC REG (Nkrumah et al., 2004). The RFI was defined as the difference between the actual and the expected DMI using the following model (Lancaster et al., 2014):


$$\mathrm{DMI}=\beta_{0}+\beta_{1}\mathrm{MBW}+\beta_{2}\;\mathrm{ADG}+\varepsilon$$

in which *β*_0_ is the y-intercept, *β*_1_ is the regression coefficient of MBW, *β*_2_ is the regression coefficient of ADG, and ε is the RFI. RFI SDs above and below the mean were used to divide heifers into high (>0.5 SD) group and low RFI (<0.5 SD) group (Nkrumah et al., 2004).

Finally, for all heifers, the five maximum RFI values and the five minimum RFI values were selected to slaughter. The duodenum, jejunum, and ileum contents were collected in plastic sterile containers (Lopes et al., 2019) at slaughter, and frozen immediately in liquid nitrogen, then stored at −80°C until subsequent microbial DNA extraction and metabolomic analysis. The RFI values and animal performance are shown in Table 1 and Supplementary Tables 2, 3.

**Table 1:** Animal performance according to residual feed intake (RFI) groups.

Items^1^	HRFI	LRFI	SEM^2^	*p*^3^
No. animals	5	5	−	−
Initial BW, kg	421.44	416.5	7.09	0.64
DMI, kg/d	9.53	8.06	0.42	0.04
ADG, kg/d	0.92	1.10	0.11	0.26
RFI, kg/d	1.00	−1.45	0.19	<0.01

1 HRFI, High residual feed intake; LRFI, low residual feed intake; DMI: dry matter intake; and ADG, average daily gain.

2 SEM, Standard error of the mean.

3 Value of *p* are derived using a Student’s *t*-test to assess the diferences between the HRFI and LRFI groups.

### DNA Extraction, 16S rRNA Gene Amplification, and Sequencing

One ileum sample was damaged from the HRFI group and one ileum sample was damaged from the LRFI group. Therefore, 16S rRNA and metabolomic analysis were only performed for the remaining 28 SI contents. Microbial DNA was extracted from the duodenum, jejunum, and ileum samples using an E.Z.N.A.® soil DNA Kit (Omega Bio-tek, Norcross, GA, United States) following the manufacturer’s protocols. Subsequently, the amplification and sequencing process of the 16S rRNA gene was described by Liu et al. (2019).

### Sequence Processing and Analysis

Raw sequences were quality-filtered using fastp version 0.20.0 (Chen et al., 2008) and merged by FLASH version 1. 2. 7 (Magoč and Salzberg, 2011) with the following criteria: (1) The 300 bp reads were truncated at any site receiving an average quality score of <20 over a 50-bp sliding window, and truncated reads shorter than 50 bp, as well as reads containing ambiguous characters, were discarded; (2) only overlapping sequences longer than 10 bp were assembled according to their overlapped sequence (maximum error ratio = 0.2); and (3) sequences of each sample were separated according to barcodes (exactly matching) and primers (allowing two nucleotide mismatches). Operational taxonomic units (OTUs) at 97% similarity were clustered using UPARSE version 7.1, with a confidence threshold of 0.70, and the taxonomy of each OTU representative sequence was analyzed using the RDP Classifier version 2.2 against the Silva 132/16S_bacteria database (Wang et al., 2007; Quast et al., 2013). Chimeric sequences were identified and removed using a novel “greedy” algorithm (Stackebrandt and Goebel, 1994; Edgar, 2013). The following analyses were performed on the Majorbio I Sanger Cloud Platform.^1^ Alpha diversity was assessed by MOTHUR version v.1.30.1 (Schloss et al., 2009). Bar graphs were constructed using the “vegan” package in R (Oksanen et al., 2010). Beta-diversity was estimated by computing the Bray–Curtis distance, calculated as similarities (ANOSIM) (999 permutations), and visualized through principal coordinate analysis (PCoA) by the “vegan” package in R (Oksanen et al., 2015). Significantly different bacteria at the phylum, family, and genus levels between the HRFI group and the LRFI group were identified by Student’s *t*-test and false discovery rate (FDR) multiple check calibration using the “stats” package in R, as well as the “scipy” package in python (Jones et al., 2001; R Core Team, 2013; Parks et al., 2014).

### Metabolomic Processing

All SI samples were analyzed using the LC–MS platform (Thermo Ultimate 3000LC, Q Exactive; ThermoFisher Scientific, Waltham, MA, United States). Briefly, 50 mg samples were weighed accurately, then 400 μl of methanol/water (4:1 v/v) was used to extract the metabolites. The mixture was allowed to settle at −20°C and treated by a high throughput tissue grinder (Wonbio-96, Shanghai Wanbo Biotechnology Co., Ltd., Shanghai, China) for 6 min at 50 Hz, followed by vortexing for 30 s and ultrasound disruption at 40 kHz for 30 min at 5°C. The samples were placed at −20°C for 30 min. The samples were then centrifuged at 13,000 × *g* for 15 min at 4°C, and the supernatant was used for LC–MS/MS analysis. Chromatographic separation of the metabolites was carried out using an ExionLC™AD system (AB Sciex, Framingham, MA, United States) equipped with an ACQUITY UPLC HSS T3 column (100 × 2.1 mm i.d., 1.8 μm particle size; Waters, Milford, MA, United States). Mobile phases consisted of A (0.1% formic acid in water) and B (acetonitrile 50% and isopropyl alcohol 50% with 0.1% formic acid). The gradient of the mobile phase (A:B) consisted of the following: 0–3 min, 95%:5%–80%:20%; 3–9 min, 80%:20%–5%:95%; 9–13 min, 5%:95%–5%:95%; 13.0–13.1 min, 5%:95%–95%:5%; and 13.1–16.0 min, 95%:5%–95%:5% to equilibrate the system. The UPLC system was coupled to a quadrupole time-of-flight mass spectrometer (Triple TOFTM5600+, AB Sciex) equipped with an electrospray ionization (ESI) source. The optimal conditions were as follows: source temperature, 500°C; curtain gas (CUR), 30 psi; both Ion Source GS1 and GS2, 50 psi; ion-spray voltage floating (ISVF), −4,000 V in negative mode and 5,000 V in positive mode, respectively; declustering potential, 80 V; collision energy (CE), 20–60 V rolling for MS/MS. To test the repeatability of the system, quality control (QC) samples prepared by mixing equal volumes of all ruminal liquid were injected at regular intervals.

### Metabolomics Data Analysis

After UPLC-TOF/MS analyses, the raw data were first imported into Progenesis QI 2.3 (Nonlinear Dynamics, Waters) for baseline filtering, peak detection, and alignment. A data matrix of retention time, mass charge ratio, and peak intensity was generated by the preprocessing results. At least 50% of the metabolic features of samples were retained. After filtering, the vacancy values were filled (the minimum value in the original matrix), and each metabolic feature was normalized by sum. After the internal standard for data QC (reproducibility) was analyzed and data were discarded if the relative SD (RSD) of QC was >30%, the statistical analysis was performed on log10 transformed data to identify significant differences in metabolite levels between the HRFI and LRFI groups. The mass spectra of these metabolic features were used to search biochemical databases such as the Human metabolome database (HMDB)^2^ and the Metlin database.^3^ All data were visualized between the HRFI and LRFI groups using principal component analysis (PCA), followed by orthogonal partial least squares discriminant analysis OPLS-DA with Student’s *t*-test and the following screening criteria: variable importance in the projection (VIP) values >1.0, difference multiple fold change (FC) > 1.0 or FC < 1.0 and *p* < 0.05 to obtain significantly differentially abundant metabolites between the LRFI and HRFI groups. Moreover, significantly differentially abundant metabolites were analyzed for abundance pattern clustering using the “gplots” package in R (Warnes et al., 2016). The impact of the RFI phenotype on metabolic pathways and metabolite set enrichment was analyzed using the “stats” package in R and the “scipy” package in python, respectively (Jones et al., 2001; R Core Team, 2013); and the significantly enriched KEGG metabolic pathway was obtained by *q* value (false discovery rate; FDR) <0.05. The correlations among significantly differentially abundant metabolites, predominant SI bacteria, and RFI phenotype were assessed using Spearman’s correlation analysis in the “pheatmap” package in R (Kolde, 2012).

## Results

### Animal Performance

As shown in Table 1, at the beginning of the experiment, the HRFI group and the LRFI group were not different in body weight (*p* = 0.64). However, the LRFI group had a lower DMI (*p* = 0.04) and a lower RFI value (*p* < 0.01) compared with the HRFI group.

### Sequencing, Alpha Diversity, and Beta Diversity

**Figure 1:**
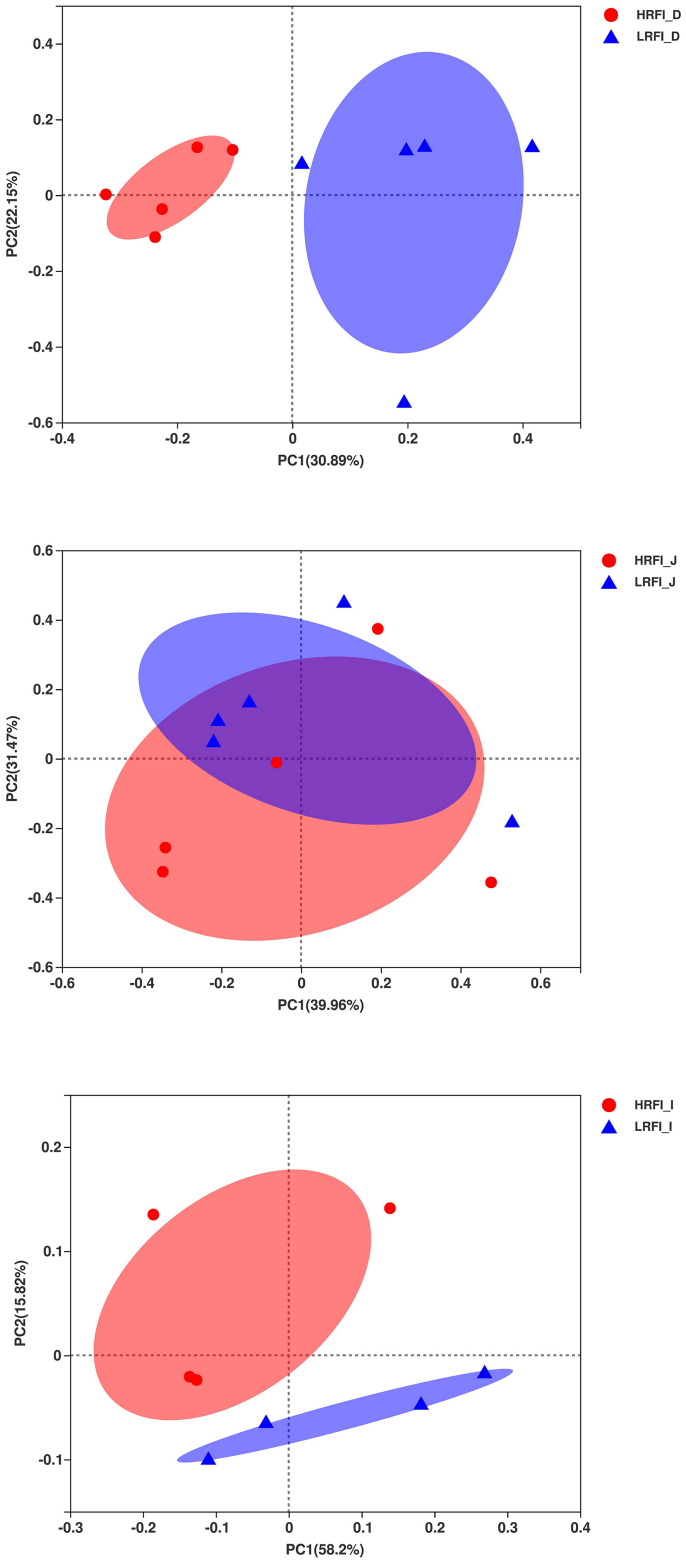
Principal coordinate analysis (PCoA) of the duodenum, jejunum, and ileum bacterial communities.

In total, 448,612; 478,572; and 496,803 raw bacterial sequences were obtained from the duodenum, jejunum, and ileum samples, respectively. After quality control to an equal sequencing depth (286,770; 337,100; or 46,378 reads per sample of the duodenum, jejunum, ileum, respectively) and clustering, we obtained 3,542; 1,589; and 1,030 OTUs at a 97% similarity level, which were assigned to 40, 28, and 22 phyla; 91, 57, and 38 classes; 245, 120, and 83 orders; 415, 208, and 144 families; and 912, 463, and 308 genera in the duodenum, jejunum, and ileum samples, respectively. Good’s coverage after normalization for the duodenum, jejunum, and ileum samples were >99.52%, 99.68%, and 99.78% for the bacterial community, respectively, indicating good sequencing coverage for the samples. Chao1’s richness, Shannon’s diversity, and Simpson’s diversity of Alpha diversity demonstrated that the bacterial community of the duodenum, jejunum, and ileum samples did not vary between the RFI groups (*p* > 0.05), except for Shannon’s diversity of the ileum (*p* = 0.04; Supplementary Table 4). Furthermore, we performed a Bray–Curtis dissimilarity analysis of the microbiota for the HRFI and LRFI groups and visualized using PCoA plots as shown in Figure 1. We found that the duodenum and ileum microbiota show clear separation, except for jejunum bacteria, indicating that the RFI phenotype influences the composition of the duodenum and ileum, respectively.

### Bacteria Abundance

**Figure 2:**
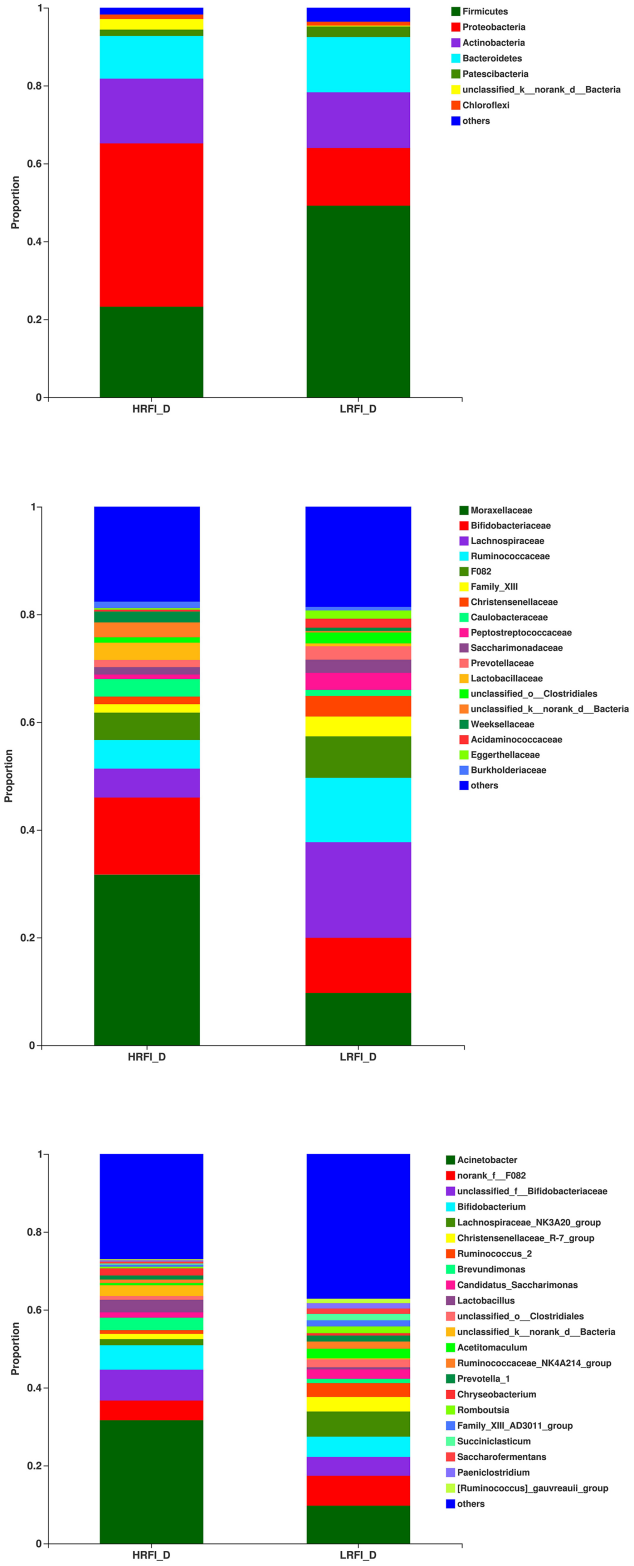
The average proportion of the most dominant duodenum bacteria phyla, families, and genera (relative abundance >1% for all samples).

In the duodenum, the phyla with relative abundances >10% were Firmicutes (23.22%, 49.16%), Proteobacteria (41.95%, 14.84%), Actinobacteria (16.60%, 14.28%), and Bacteroidetes (10.96%, 14.20%) for the HRFI group and LRFI group, respectively. At the family level, bacterial families with relative abundance >10% were *Moraxellaceae* (31.68%, 9.70%), *Bifidobacteriaceae* (14.32,10.26%), *Lachnospiraceae* (5.36%, 17.73%), and *Ruminococcaceae* (5.33,11.96%) in the HRFI group and LRFI group, respectively. The genera of bacteria with relative abundances >5% were *Acinetobacter* (31.64%, 9.68%), *norank_f_F082* (5.06%, 7.70%), *unclassified_f_Bifidobacteriaceae* (7.91%, 4.84%), *Bididobacterium* (6.27%, 5.19%), and *Lachnospiraceae_NK3A20_group* (1.57%, 6.45%) in the HRFI group and LRFI group, respectively (Figure 2).

**Figure 3:**
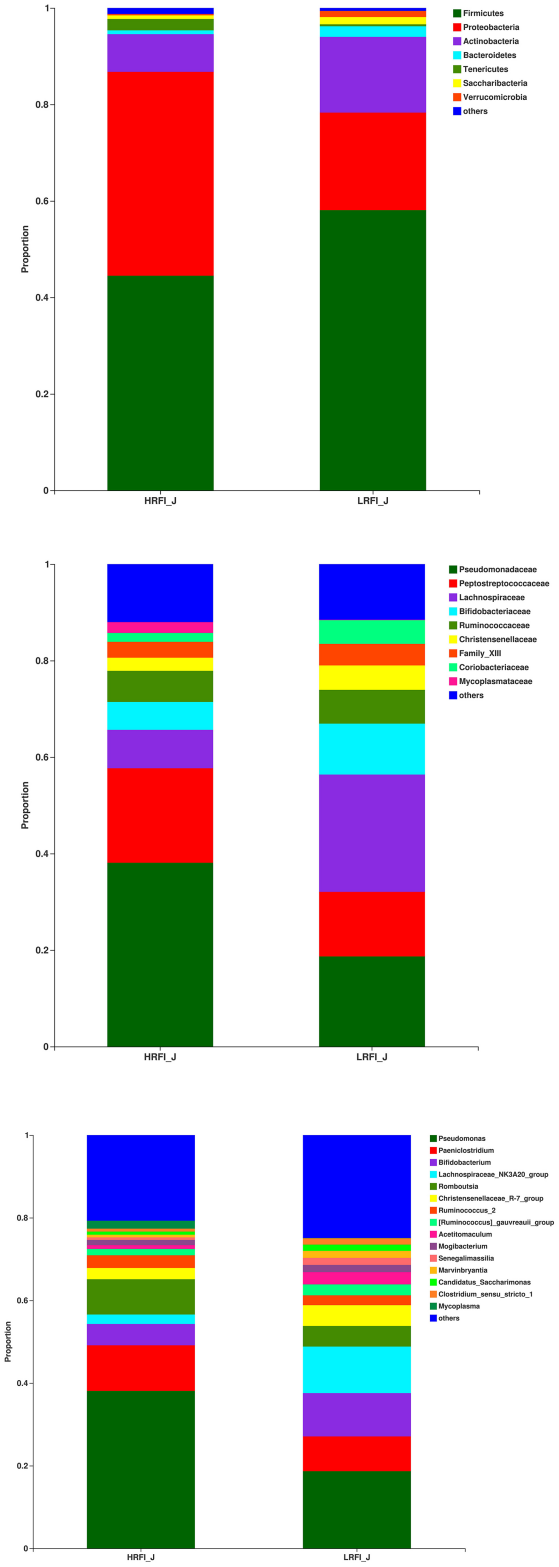
The average proportion of the most dominant jejunum bacteria phyla, families, and genera (relative abundance >1% for all samples).

In the jejunum, the phyla with relative abundances >10% were Firmicutes (44.49%; 58.09%), Proteobacteria (42.27%; 20.21%), and Actinobacteria (7.79%; 15.69%) for the HRFI group and LRFI group, respectively. At the family level, bacterial families with a relative abundance >10% were *Pseudomonadacese* (38.09%, 18.68%), *Peptostreptococcaceae* (19.60%, 13.40%), *Lachnospiraceae* (7.95%, 24.29%), and *Bifidobacteriaceae* (5.78%, 10.56%) in the HRFI group and LRFI group, respectively. The genera of bacteria with relative abundances >5% were *Pseudomonadacese* (38.09%, 18.68%), *Paeniclostridium* (11.05%, 8.42%), *Bifidobacterium* (5.15%, 10.46%), *Lachnospiraceae_NK3A20_group* (2.29%, 11.27%), *Romboutsia* (8.54%, 4.98%), and *Christensenellaceae_R-7_group* (2.74%, 5.05%) in the HRFI group and LRFI group, respectively (Figure 3).

**Figure 4:**
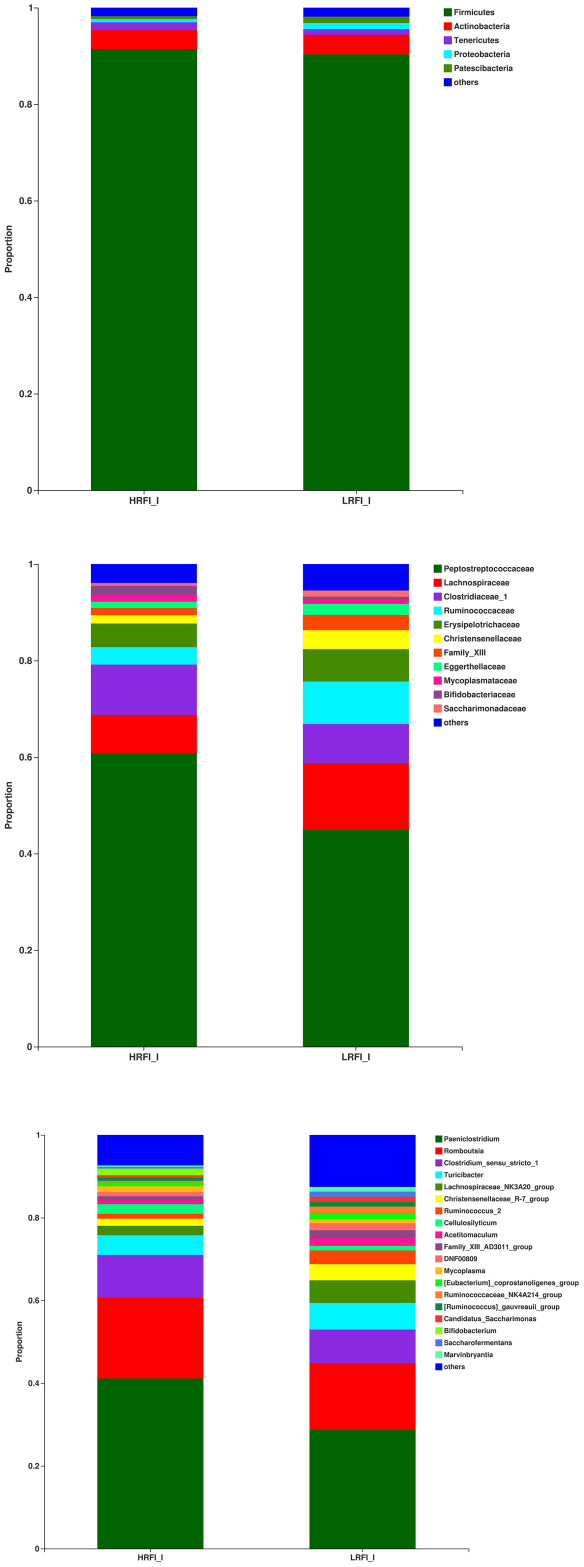
The average proportion of the most dominant ileum bacteria phyla, families, and genera (relative abundance >1% for all samples).

In the ileum, the phyla with relative abundances >10% were Firmicutes (91.41%; 90.31%), and Actinobacteria (3.93%, 4.06%) for the HRFI group and LRFI group, respectively. At the family level, bacterial families with a relative abundance >10% were *Peptostreptococcaceae* (60.71%, 44.91%), *Lachnospiraceae* (8.06%, 13.82%), *Clostridiaceae_1* (10.39%, 8.12%) in the HRFI group and LRFI group, respectively. The genera of bacteria with relative abundances >5% were *Paeniclostridium* (41.12%, 28.72%), *Romboutsia* (19.52%, 16.12%), *Clostridium_sensu_stricto_1* (10.33%, 8.09%), *Turicibacter* (4.78%, 6.48%), *Lachnospiraceae_NK3A20_group* (2.27%, 5.43%) in the HRFI group and LRFI group, respectively (Figure 4).

### Significantly Differentially Abundant SI Bacteria

**Figure 5:**
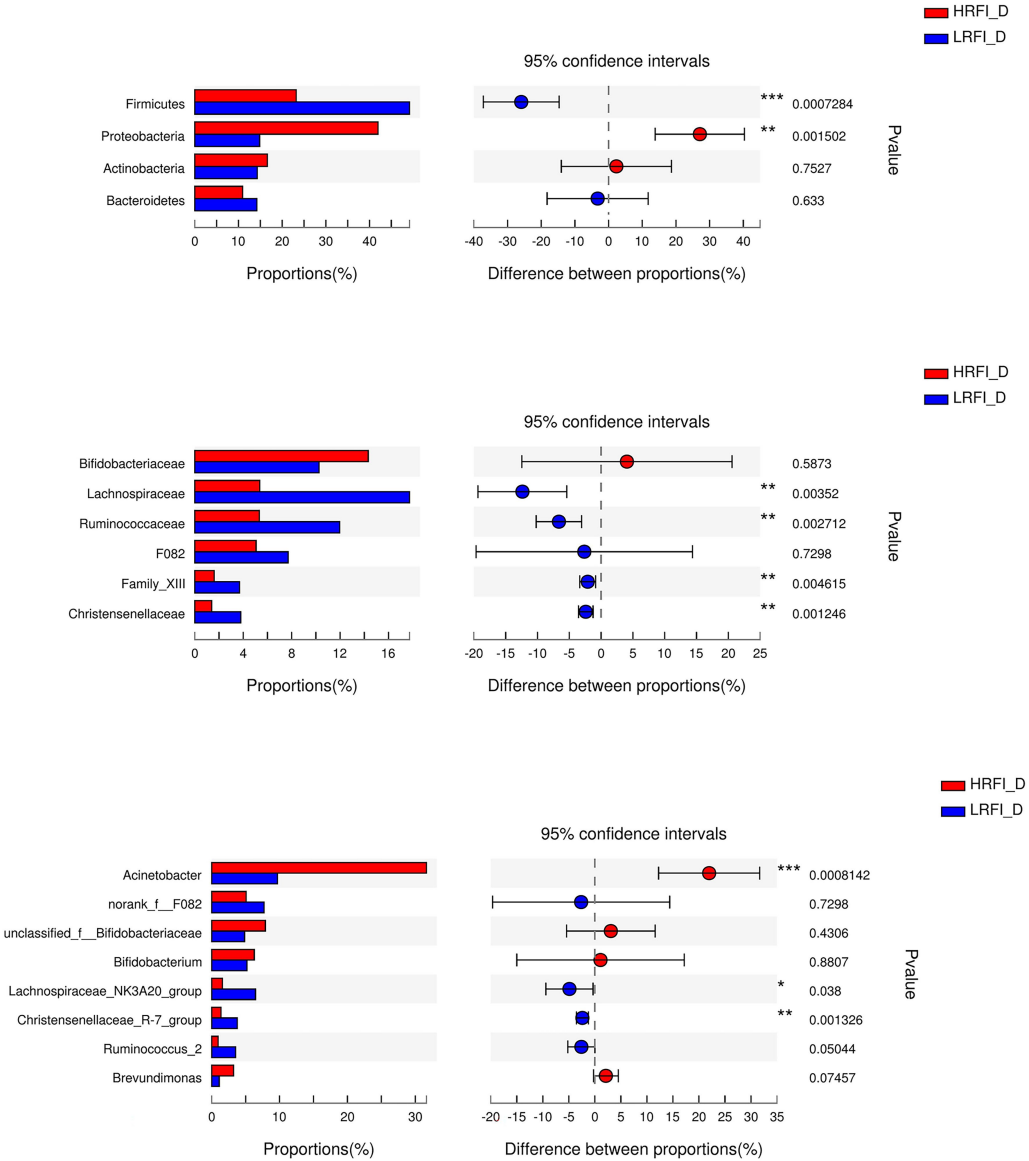
Significantly differential abundant phyla, families, and genera (relative abundance >1%) with the duodenum bacteria. Positive and negative differences indicate a greater abundance in the HRFI group and LRFI group, respectively. *Represents 0.01 < *p* < 0.05; ** represents 0.001 < *p* < 0.01; and *** represents *p* < 0.001.

**Figure 6:**
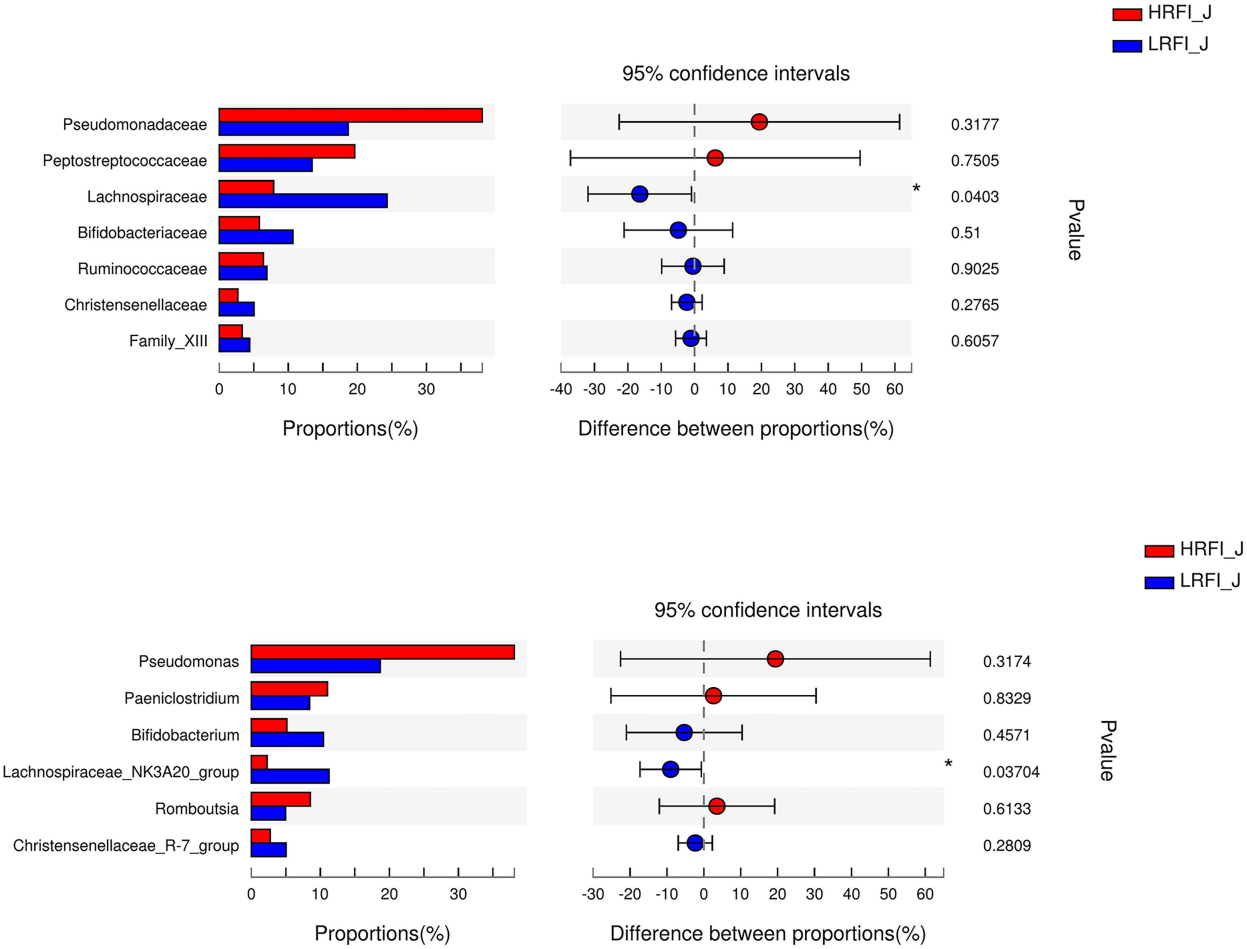
Significantly differential abundant families, and genera (relative abundance >1%) with the jejunum bacteria. Positive and negative differences indicate a greater abundance in the HRFI group and LRFI group, respectively. *Represents 0.01 < *p* < 0.05.

**Figure 7:**
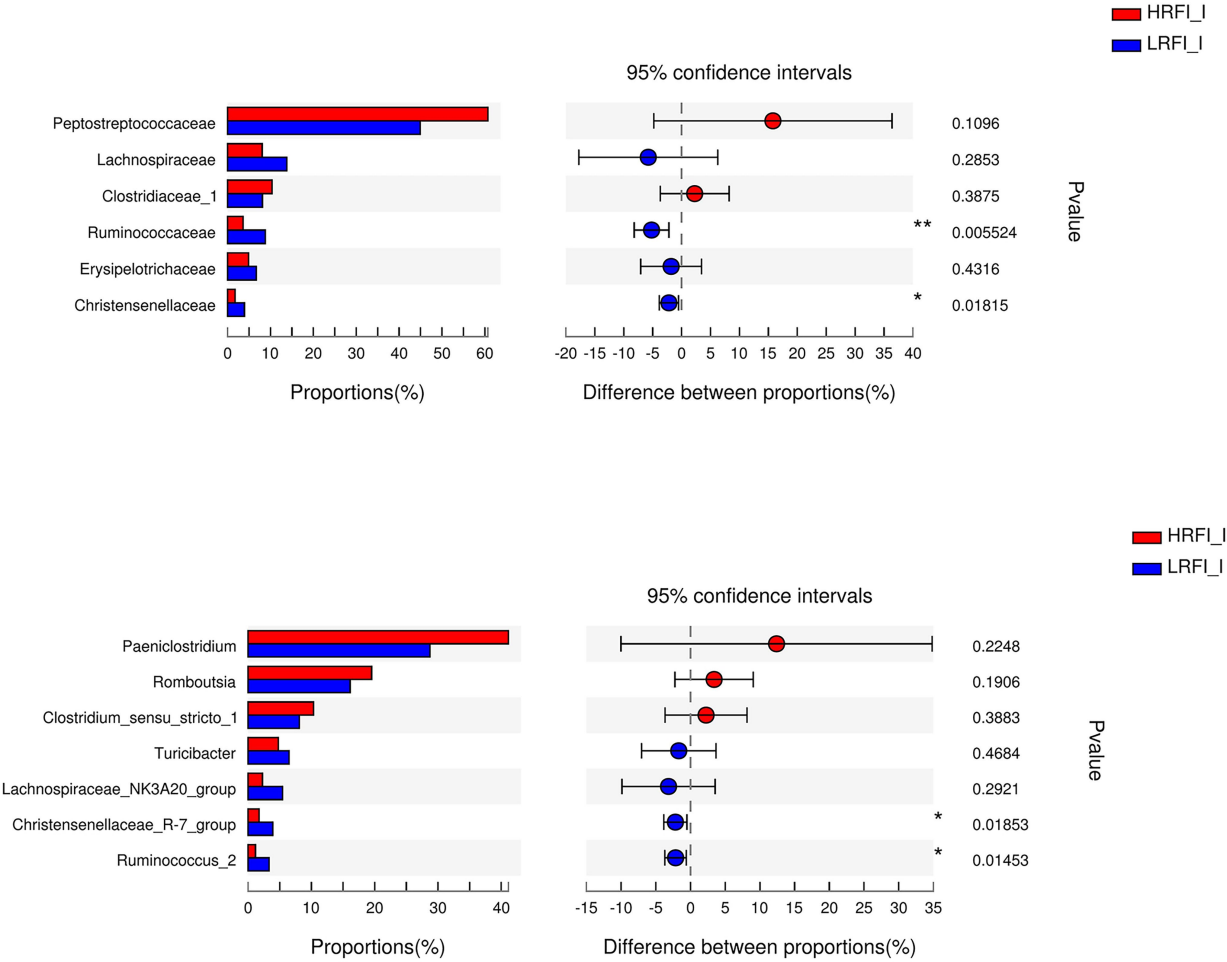
Significantly differential abundant families, and genera (relative abundance >1%) with the ileum bacteria. Positive and negative differences indicate a greater abundance in the HRFI group and LRFI group, respectively. *Represents 0.01 < *p* < 0.05 and ** represents 0.001 < *p* < 0.01.

As shown in Figure 5, in the duodenum, at the phylum level, the relative abundances of Firmicutes (23.22%, 49.16%) and Proteobacteria (41.95%, 14.84%) was higher in the LRFI group and HRFI group, respectively (*p* < 0.01). At the family level, the relative abundances of *Lachnospiraceae* (5.36%, 17.73%), *Ruminococcaceae* (5.33%, 11.96%), *Family_XIII* (1.60%, 3.70%), and *Christensenellaceae* (1.39%, 3.80%) were higher in the LRFI group (*p* < 0.01). At the genus level, the relative abundances of *Lachnospiraceae_NK3A20_group* (1.57%, 6.45%; *p* < 0.05), and *Christensenellaceae_R-7_group* (1.37%, 3.77%; *p* < 0.01) were higher in the LRFI group, whereas the HRFI group had a higher abundance of *Acinetobacter* (32.12%, 11.19%; *p* < 0.01). As shown in Figure 6, in the jejunum, the relative abundances of family *Lachnospiraceae* (7.95%, 24.29%) and genus *Lachnospiraceae_NK3A20_group* (2.29%, 11.27%) were higher in the LRFI group (*p* < 0.05). In the ileum, the relative abundances of families *Ruminococcaceae* (3.62%, 8.81%; *p* < 0.01) and *Christensenellaceae* (1.74%, 3.93%; *p* < 0.05) were higher in the LRFI group; the relative abundance of genus *Christensenellaceae_R-7_group* (1.73%, 3.91%) and *Ruminococcus_2* (1.16%, 3.31%) were higher (*p* < 0.05) in the LRFI group (Figure 7).

### Metabolomic Profiling

#### Sample Quality Control

**Figure 8:**
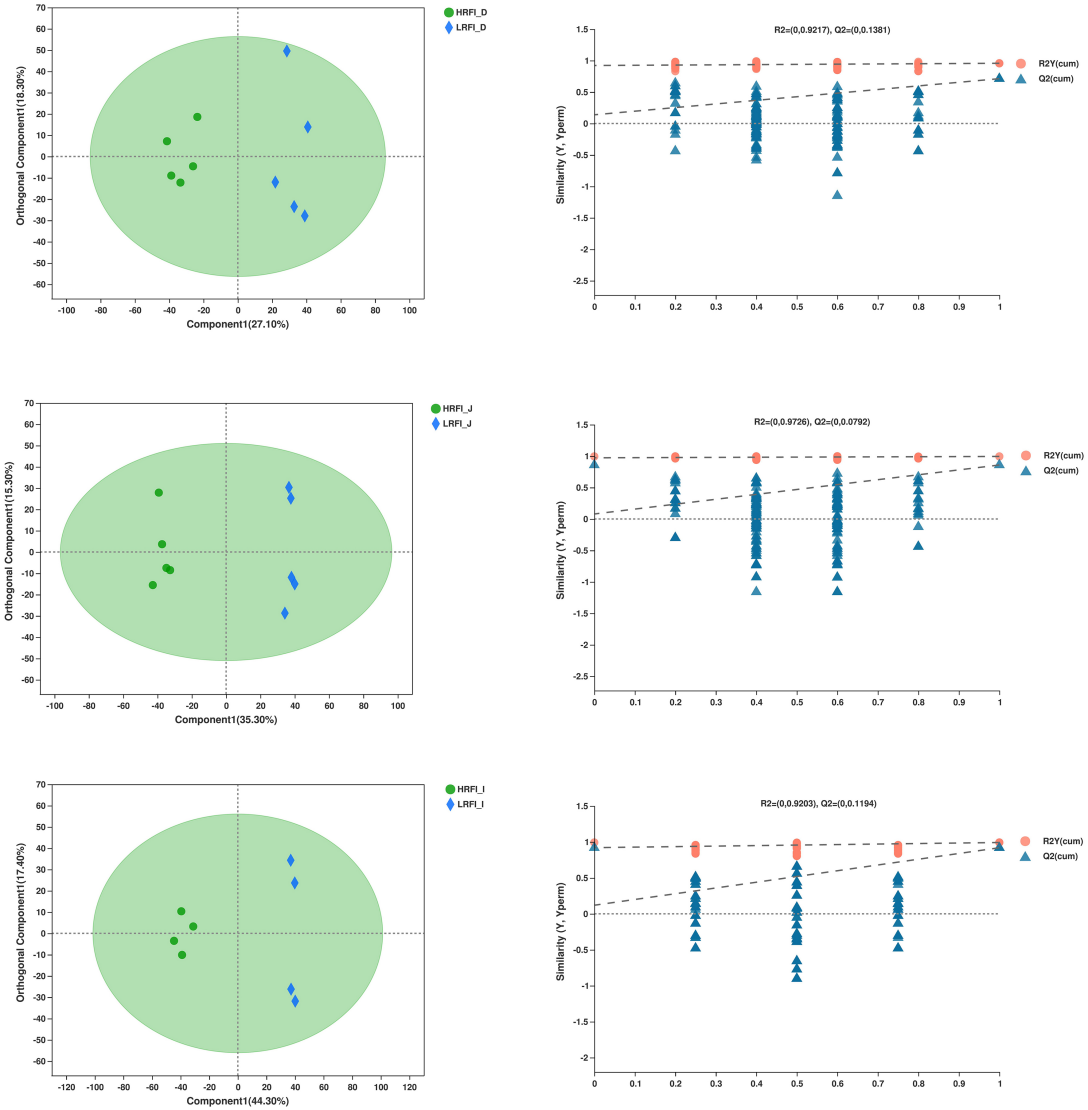
Orthogonal partial least squares discriminant analysis (OPLS-DA) plot of the duodenum, jejunum, ileum bacterial metabolites.

The overlap of the total ion chromatogram of the QC samples in the positive (A) and negative (B) ion modes is shown in Supplementary Figure 1, which confirmed the stability and reproducibility of the data. Figure 8 shows the OPLS-DA score plot of duodenum, jejunum, ileum between the HRFI group and LRFI group, all the samples were within the 95% Hotelling T2 ellipse, and the permutation test with a better range of *R*^2^-values from 0.920 to 0.973, indicating moderate effectiveness of the model.

#### Significantly Differentially Metabolite Analysis

The PCA provided a satisfactory separation of the data between the groups (Supplementary Figure 2). As shown in Table 2, there are 6, 10, and 16 differential metabolites between the LRFI group and HRFI group in the duodenum, jejunum, and ileum, respectively. Moreover, we found that the LRFI group significantly improved protein digestion and absorption, as well as glycerophospholipid metabolism in the duodenum, jejunum, and ileum.

**Table 2:** Significantly differentially metabolites in small intestine by comparison of the LRFI and HRFI groups.^1^

Small intestine	Processes	Metabolites	Formula	VIP^2^	*p*^3^	FC^4^	Trend^5^
Duodenum	Protein digestion and absorption	L-Glutamate	C_5_H_9_NO_4_	1.26	*p* < 0.02	1.09	Up
		L-Tyrosine	C_9_H_11_NO_3_	1.37	*p* < 0.02	1.05	Up
		Beta-alanine	C_3_H_7_NO_2_	1.48	*p* < 0.03	1.20	Up
	Glycerophospholipid metabolism	PC (18:2(9Z,12Z)/20:4(5Z,8Z,11Z,14Z))	C_46_H_80_NO_8_P	1.34	*p* < 0.03	1.11	Up
		Phosphocholine	C_5_H_14_NO_4_P	1.35	*p* < 0.03	1.14	Up
		Choline	C_5_H_13_NO	1.32	*p* < 0.04	1.06	Up
Jejunum	Protein digestion and absorption	L-Glutamate	C_5_H_9_NO_2_	1.50	*p* < 0.01	1.08	Up
		L-Serine	C_5_H_10_N_2_O_3_	1.36	*p* < 0.01	1.14	Up
		L-Methionine	C_5_H_11_NO_2_S	1.51	*p* < 0.01	1.05	Up
		L-Histidine	C_6_H_9_N_3_O_2_	1.21	*p* < 0.03	1.05	Up
		L-Aspartic acid	C_4_H_7_NO_4_	1.38	*p* < 0.01	1.13	Up
		L-Isoleucine	C_6_H_13_NO_2_	1.51	*p* < 0.01	1.04	Up
		L-Tyrosine	C_9_H_11_NO_3_	1.31	*p* < 0.01	1.03	Up
	Glycerophospholipid metabolism	PC (18:2(9Z,12Z)/20:4(5Z,8Z,11Z,14Z))	C_46_H_80_NO_8_P	1.38	*p* < 0.02	1.46	Up
		PS (18:0/20:4(8Z,11Z,14Z,17Z))	C_44_H_78_NO_10_P	1.15	*p* < 0.05	1.39	Up
		Choline	C_5_H_13_NO	1.48	*p* < 0.01	1.13	Up
lleum	Protein digestion and absorption	L-Glutamate	C_5_H_9_NO_4_	1.16	*p* < 0.04	1.06	Up
		L-Serine	C_3_H_7_NO_3_	1.25	*p* < 0.01	1.15	Up
		Beta-alanine	C_3_H_7_NO_2_	1.18	*p* < 0.01	1.07	Up
		L-Aspartic acid	C_4_H_7_NO_4_	1.21	*p* < 0.01	1.09	Up
		L-Lysine	C_6_H_14_N_2_O_2_	1.21	*p* < 0.01	1.43	Up
	Glycerophospholipid metabolism	PC (18:2(9Z,12Z)/20:4(5Z,8Z,11Z,14Z))	C_46_H_80_NO_8_P	1.23	*p* < 0.03	1.08	Up
		LysoPC (20:4(8Z,11Z,14Z,17Z))	C_28_H_50_NO_7_P	1.31	*p* < 0.01	1.34	Up
		LysoPC (20:4(5Z,8Z,11Z,14Z))	C_28_H_50_NO_7_P	1.25	*p* < 0.01	1.07	Up
		LysoPC (20:2(11Z,14Z))	C_28_H_54_NO_7_P	1.17	*p* < 0.04	1.16	Up
		LysoPC (18:1(11Z))	C_26_H_52_NO_7_P	1.37	*p* < 0.01	1.10	Up
		LysoPC (18:1(9Z))	C_26_H_52_NO_7_P	1.41	*p* < 0.01	1.13	Up
		LysoPC (16:0)	C_24_H_50_NO_7_P	1.23	*p* < 0.02	1.09	Up
		LysoPC (18:0)	C_26_H_54_NO_7_P	1.15	*p* < 0.04	1.11	Up
		LysoPC (22:0)	C_30_H_62_NO_7_P	1.41	*p* < 0.01	1.43	Up
		Choline	C_5_H_13_NO	1.39	*p* < 0.01	1.09	Up
		Phosphocholine	C_5_H_14_NO_4_P	1.40	*p* < 0.01	1.89	Up

1 LRFI, Low residual feed intake and HRFI, high residual feed intake.

2 VIP, Variable importance in the projection.

3 Value of *p* (false discovery rate) are derived using a Student’s *t*-test to assess the diferences between the HRFI group and the LRFI group; significance was considered at *p* < 0.05.

4 FC, Fold change.

5 Up, upregulated and down, downregulated.

#### Correlation Analysis Among the Predominant Genera Bacteria, Significantly Differentially Metabolites, and the RFI Phenotype

**Figure 9:**
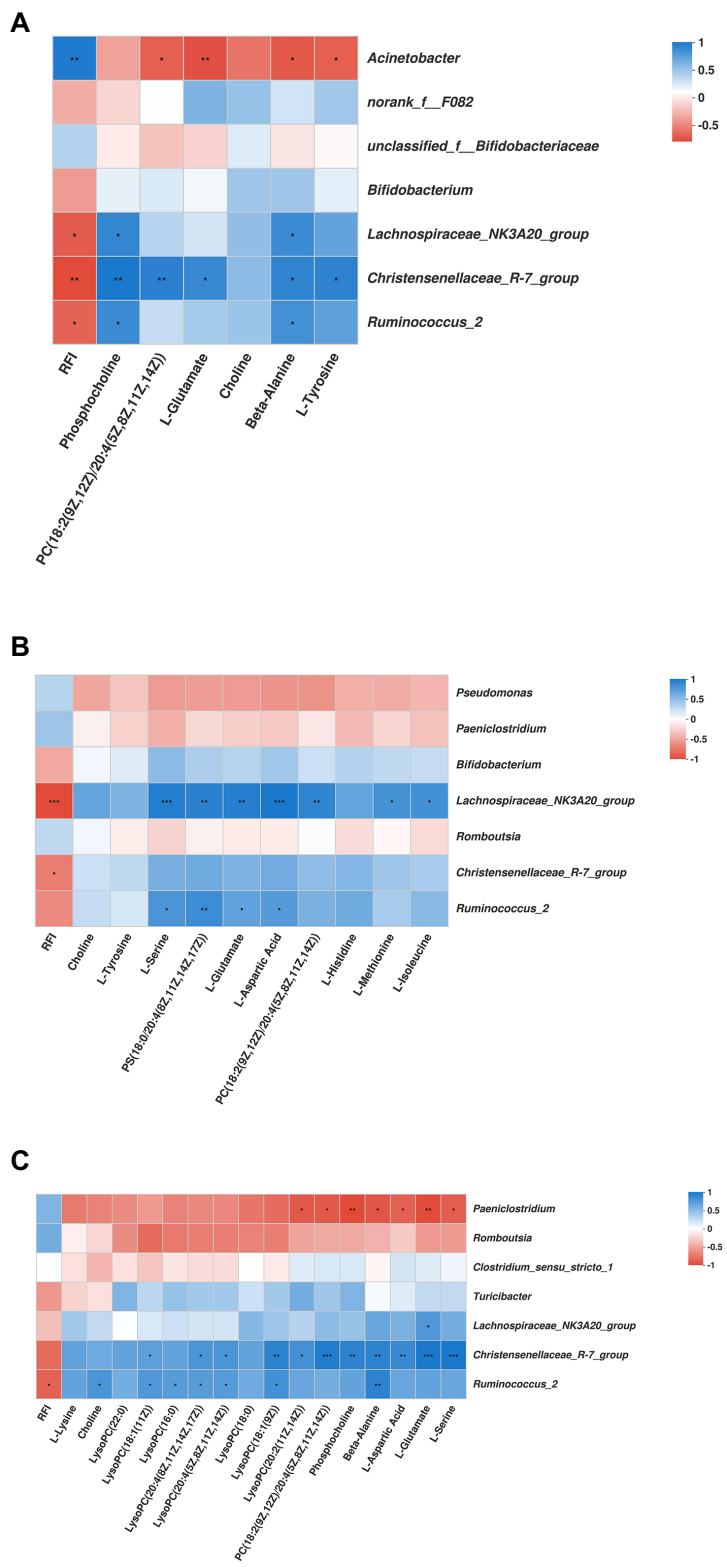
Correlation analysis among the predominant genera duodenum **(A)**, jejunum **(B)**, ileum **(C)** microbiota bacteria, the significantly differential metabolites, and the RFI. Cells are colored based on Spearman’s correlation coefficient: blue represents a positive correlation; red represents a negative correlation. *Represents 0.01 < *p* ≤ 0.05; ** represents 0.001 < *p* ≤ 0.01; and *** represents *p* ≤ 0.001.

As shown in Figure 9, in the duodenum, the genus *Acinetobacter* was positively associated with RFI (*r* = 0.81, *p* < 0.01), while negatively associated with PC [18:2(9Z,12Z)/20:4(5Z,8Z,11Z,14Z)] (*r* = −0.70, *p* < 0.05), L-Glutamate (*r* = −0.77, *p* < 0.01), beta-Alanine (*r* = −0.73, *p* < 0.05), and L-Tyrosine (*r* = −0.70, *p* < 0.05). The genus *Lachnospiraceae_NK3A20_group* was negatively associated with RFI (*r* = −0.72, *p* < 0.05), while positively associated with phosphocholine (*r* = 0.73, *p* < 0.05), and beta-Alanine (*r* = 0.71, *p* < 0.05). The genus *Christensenellaceae_R-7_group* was negatively associated with RFI (*r* = −0.79, *p* < 0.01), while positively associated with phosphocholine (*r* = 0.82, *p* < 0.01), PC [18:2(9Z,12Z)/20:4(5Z,8Z,11Z,14Z)] (*r* = 0.77, *p* < 0.01), L-Glutamate (*r* = 0.72, *p* < 0.05), beta-Alanine (*r* = 0.75, *p* < 0.05), and L-Tyrosine (*r* = 0.76, *p* < 0.05). The genus *Ruminococcus_2* was negatively associated with RFI (*r* = −0.69, *p* < 0.05), while positively associated with phosphocholine (*r* = 0.71, *p* < 0.05), and beta-Alanine (*r* = 0.67, *p* < 0.05). In the jejunum, the genus *Lachnospiraceae_NK3A20_group* was negatively associated with RFI (*r* = −0.92, *p* < 0.001), while positively associated with L-Serine (*r* = 0.88, *p* < 0.001), PS [18:0/20:4(8Z,11Z,14Z,17Z)] (*r* = 0.86, *p* < 0.01), L-Glutamate (*r* = 0.87, *p* < 0.01), and L-Aspartic Acid (*r* = 0.92, *p* < 0.001), PC [18:2(9Z,12Z)/20:4(5Z,8Z,11Z,14Z)] (*r* = 0.83, *p* < 0.05), L-Methionine (*r* = 0.75, *p* < 0.05), and L-Isoleucine (*r* = 0.76, *p* < 0.05). The genus *Christensenellaceae_R-7_group* was negatively associated with RFI (*r* = −0.66, *p* < 0.01). The genus *Ruminococcus_2* was positively associated with L-Serine (*r* = 0.72, *p* < 0.05), PS [18:0/20:4(8Z,11Z,14Z,17Z)] (*r* = 0.78, *p* < 0.01), L-Glutamate (*r* = 0.64, *p* < 0.05), and L-Aspartic Acid (*r* = 0.69, *p* < 0.05). In the ileum, the genus *Paeniclostridium* was negatively associated with LysoPC [20:2(11Z,14Z)] (*r* = −0.79, *p* < 0.05), PC [18:2(9Z,12Z)/20:4(5Z,8Z,11Z,14Z)] (*r* = −0.79, *p* < 0.05), phosphocholine (*r* = −0.86, *p* < 0.01), beta-Alanine (*r* = −0.81, *p* < 0.05), L-Aspartic Acid (*r* = −0.74, *p* < 0.05), L-Glutamate (*r* = −0.86, *p* < 0.01), and L-Serine (*r* = −0.76, *p* < 0.05). The genus *Lachnospiraceae_NK3A20_group* was positively associated with L-Glutamate (*r* = 0.76, *p* < 0.05). The genus *Christensenellaceae_R-7_group* was positively associated with LysoPC [18:1(11Z)] (*r* = 0.71, *p* < 0.05), LysoPC [20:4(8Z,11Z,14Z,17Z)] (*r* = 0.79, *p* < 0.05), LysoPC [20:4(5Z,8Z,11Z,14Z)] (*r* = 0.79, *p* < 0.05), LysoPC [18:1(9Z)] (*r* = 0.91, *p* < 0.01), LysoPC [20:2(11Z,14Z)] (*r* = 0.81, *p* < 0.05), PC [18:2(9Z,12Z)/20:4(5Z,8Z,11Z,14Z)] (*r* = 0.95, *p* < 0.001), phosphocholine (*r* = 0.91, *p* < 0.01), beta-Alanine (*r* = 0.91, *p* < 0.01), L-Aspartic Acid (*r* = 0.86, *p* < 0.01), L-Glutamate (*r* = 0.98, *p* < 0.001), and L-Serine (*r* = 0.98, *p* < 0.001). The genus *Ruminococcus_2* was negatively associated with RFI (*r* = −0.74, *p* < 0.05), while positively associated with choline (*r* = 0.79, *p* < 0.05), LysoPC [18:1(11Z)] (*r* = 0.76, *p* < 0.05), LysoPC (16:0) (*r* = 0.74, *p* < 0.05), LysoPC [20:4(8Z,11Z,14Z,17Z)] (*r* = 0.74, *p* < 0.05), LysoPC [20:4(5Z,8Z,11Z,14Z)] (*r* = 074, *p* < 0.05), LysoPC [18:1(9Z)] (*r* = 0.81, *p* < 0.05), and beta-Alanine (*r* = 0.91, *p* < 0.01).

## Discussion

### Animal Performance

As expected, the LRFI heifers consumed less feed during this experiment, consistent with the result on steers (Welch et al., 2020). Improving feed efficiency by decreasing feed cost means the greatest profitability of the beef cattle production system (Lancaster et al., 2009). Therefore, we should select LRFI cattle to consume less feed without affecting ADG, resulting in maximized profitability for the beef cattle industry (Herd et al., 2003).

### Bacterial Diversity

The diverse microorganisms that colonize the digestive tract of beef cattle play a vital role in the host’s digestion and absorption of nutrients, promotion of immunity, regulation of behavior, and maintenance of intestinal homeostasis. In the present study, we found that the diversity of the bacterial communities in the ileum was significantly higher in the LRFI group than in the HRFI group. This might be explained by previous observations that higher intestinal microbial diversity and richness result in a more stable and functionally complete intestinal ecosystem, which means that animals have better adaptability and higher productivity (Firkins and Yu, 2015; Huws et al., 2018). However, we found no differences in diversity and richness of duodenum and jejunum with different RFI phenotypes, previous studies were similar to our result (Li and Guan, 2017; Perea et al., 2017; Paz et al., 2018; Freetly et al., 2020; Lopes et al., 2021). This implies that efficiency status does not depend on a large-scale restructuring of the entire microbial community, but might be dependent on differences in a few key taxa (Perea et al., 2017). Additionally, some studies also observed no differences in rumen microbial diversity of cattle with different RFI phenotypes (Paz et al., 2018; Clemmons et al., 2019; Lopes et al., 2021). These results might indicate that the diversity of the microbial community is not necessarily related to the RFI phenotype of animals. Because of current conflicting results, in the future, more research is needed to confirm these results. Moreover, the structure of the GIT bacterial community has been proven to be host-specific, which might lead to large differences in the diversity and richness of the GIT bacterial community (Huttenhower et al., 2012; Donaldson et al., 2016).

### Significantly Differentially Bacteria

In this study, we found that the SI bacterial community structures in duodenum, jejunum, and ileum of heiferes with different RFI phenotypes were significantly different. Additionally, the duodenum, jejunum, and ileum of heifers with different RFI phenotypes might have a similar “core” bacterial microbiota community structure, as well as some specific microbiota (Myer et al., 2016a; Perea et al., 2017; Freetly et al., 2020). Differences were observed for some SI taxa, specifically, the Firmicutes and Proteobacteria phyla were consistent with a previous study of beef cattle (Freetly et al., 2020) and lamb (Perea et al., 2017), respectively. In the present study, Lachnospiraceae was found in greater abundance in the duodenum, and jejunum of the most efficient heifers; as well as *Ruminococcaceae* in the duodenum, and ileum. Coincident with our results, Shabat et al. (2016) reported that dairy cows with an LRFI phenotype also had greater levels of Lachnospiraceae. Moreover, Gagen et al. (2015) observed that acetogens could be found in both the families Lachnospiraceae and *Ruminococcaceae*, which might be explained by the presence of more families Lachnospiraceae and *Ruminococcaceae* in the LRFI group: it is likely that elevated levels of Lachnospiraceae and *Ruminococcaceae* indicate a more complete fermentation and increased absorbable nutrients available to LRFI animals (Freetly et al., 2020). Additionally, *Christensenellaceae* has been identified as an indicator of a “healthy digestive system” (Goodrich et al., 2014), and is a known butyrate producer (Morotomi et al., 2012), which verified our observation that higher relative abundance of the family *Christensenellaceae* in the duodenum and ileum of the LRFI group. Members of the *Lachnospiraceae_NK3A20_group* were abundant in the SI, being present in the rumen, cecum, and fecal samples (Lopes et al., 2019), and *Ruminococcus* is found commonly within microbial communities across the GIT (Oliveira et al., 2013; Myer et al., 2016b); however, fewer studies about *Family_XIII*, *Acinetobacter*, *Lachnospiraceae_NK3A20_group*, and *Ruminococcus_2*, in the future, more studies need to be investigated.

### Significantly Differentially Metabolites

Our metabolome data revealed that the RFI significantly altered the concentrations of SI metabolites associated with protein digestion and absorption, glycerophospholipid metabolism. Additionally, correlation analysis showed that there was a certain correlation between SI microorganisms and SI differential metabolites, there is a relationship between the SI microorganisms and the SI differential metabolites. In the gut, amino acids are the degradation products of rumen-protected proteins and microbial proteins, which regulate certain metabolic pathways (Mariz et al., 2018). Notably, we observed increased levels of L-Methionine, L-Lysine, L-Isoleucine, L-Histidine, L-Glutamate, L-Aspartic Acid, Beta-Alanine, L-Serine, and L-Tyrosine in the LRFI group. Greenwood and Titgemeyer (2000) reported that for steers, corn-soybean meal or other feedstuff based diets, the first or second limiting amino acids are basically Lysine and Methionine. Moreover, Isoleucine (Swanepoel et al., 2010a,b) and Histidine (Vanhatalo et al., 1999; Greenwood and Titgemeyer, 2000) might be the third limiting amino acids, but the determination of the third limiting amino acids remains to be further studied. Archibeque et al. (2002) also confirmed methionine as a typically limiting amino acid (AA) for beef steers. Methionine plays important role in protein synthesis, DNA methylation, lipid metabolism, and antioxidant regulation (Löest et al., 2002; Zhou et al., 2016; Martinez et al., 2017). As methyl donors, rumen protected choline has been reported to improve growth performance of cattle (Bindel et al., 2000). Han et al. (2017) also observed the supplementation of methionine improved growing Holstein steers’ performance. Hussein and Berger (1995) also showed that feeding rumen-protected methionine and rumen-protected Lysine to Holstein steers improved average daily gain. Additionally, it has been reported that Lysine deficiency significantly increases anxiety in rats when stimulated by electric shock (Smriga et al., 2000). Histidine is a semi-essential amino acid in animals, and its supply has an obvious linear relationship with protein turnover of the body (Wantanee et al., 2002). Studies have observed that long-term intake of low histidine diets significantly reduces animal body weight and feed intake, reduced activity, lethargy, and even death (Cianciaruso et al., 1981). Studies have shown that histidine could maintain the stability of cell pH, maintain cell homeostasis, reducing stress (Rao et al., 2010) and protecting the heart (Obata et al., 1999). Additionally, histidine has anti-inflammatory and antioxidant effects (Feng et al., 2013). Dong et al. (2005) reported that histidine can inhibit the inflammatory response caused by oxidative stress in human intestinal epithelial cells and improve intestinal health. Meanwhile, histidine contains imidazolidyl, which can combine with Metal ions such as Cu^2+^ and Zn^2+^ to promote intestinal absorption of Cu^2+^ and Zn^2+^. Metal ions can participate in the composition of various enzymes (SOD etc.) *in vivo*. Sun et al. (2014) showed that histidine can significantly improve the antioxidant capacity of SOD in mice plasma, reduce MDA content and up-regulate the mRNA expression of CuZnSOD. L-isoleucine is a bioactive molecule involved in nutrient metabolism (Bampidis et al., 2020). Glutamate is the main oxidizing energy supply substance in the intestinal tract (Burrin et al., 2008), and it is important in intestinal antioxidant stress (Duan et al., 2014; Yin et al., 2015). Studies have found that serine is located at key nodes of multiple biological metabolic processes in the body, and plays an indispensable role in promoting cell proliferation (Newman and Maddocks, 2017), antioxidant (He et al., 2020) and immune function (Kitamoto et al., 2020). It is important to maintain animal health and normal physiological functions (Kalhan and Hanson, 2012). Additionally, carnosine, which is composed of histidine and alanine, is an endogenous functional substance and plays an important role in the body’s antioxidant function (Guiotto et al., 2005). Tyrosine is the precursor of melanin synthesis. Under the catalytic action of tyrosinase, tyrosine in animals can synthesize melanin through a series of complex biological processes. Melanin has the function of scavenging free radicals and anti-oxidation (Ye et al., 2014). Wang et al. (2018) reported that increasing the content of tyrosine in feed would lead to the increase of melanin content in the belly and back skin of tilapia. Therefore, the higher feed efficiency of LRFI group may be related to the antioxidant capacity and immune function of the upregulation of L-Methionine, L-Lysine, L-Histidine, L-Isoleucine, L-Glutamate, L-Aspartic Acid, Beta-Alanine, L-Serine, and L-Tyrosine.

It is noteworthy that we also observed alterations in the concentrations of several metabolites associated with glycerophospholipid metabolism. Choline mainly exists as lysophosphatidylcholine (LysoPCs) and phosphocholine in the body, which is important in maintaining the integrity of the cell membrane, and methyl metabolism (Blusztajn, 1998). In ruminants, it has been suggested that rumen-protected choline inhibits fat synthesis in the liver of dairy cattle during the transition period by increasing fatty acid transport and reducing the synthesis of very-low-density lipoprotein (Goselink et al., 2013). Brautigan et al. (2017) found that LysoPCs affect nutrient absorption in the intestinal tract by regulating gene expression of small intestinal epithelial cells, thus having a positive impact on the production performance of livestock. Malanka et al. (2012) also reported that LysoPCs play a regulator of immunological cell functions. Additionally, the function of phosphatidylserine in signal transduction and intercellular information transmission has been demonstrated (Nishizuka, 1984). Therefore, the upregulation of choline, phosphocholine, LysoPCs, PC [18:2(9Z,12Z)/20:4(5Z,8Z,11Z,14Z)], and PS[18:0/20:4(8Z,11Z,14Z,17Z)] might suggest increased maintain integrity of cell membrane, fatty acid transport, nutrient absorption, immune function, and signal transduction in the LRFI group to improve feed efficiency.

### Correlation Among the Predominant Bacteria, Significantly Differentially Metabolites, and the RFI Phenotype

Small intestine is the main place to nutrient digestion and absorption, the nutrients such as carbohydrate, protein, lipid after into the intestinal microbes and various enzymes under the effect of extracellular digestion, digestion products by the various transport carrier absorption of intestinal epithelial cells into the blood, and shipped to all parts of the body. Moreover, Arumugam et al. (2011) reported that some microorganisms in the intestinal tract are rich in amylase, protease and other genes to decompose carbohydrates and proteins. Marie et al. (2014) reported that the genus *Acinetobacter* plays role in opportunistic human infections, studies also have shown that glutamate, alanine, and tyrosine are play important roles in the body’s antioxidant function (Guiotto et al., 2005; Duan et al., 2014; Ye et al., 2014; Yin et al., 2015; Wang et al., 2018), which might explain why Acinetobacter was associated positively with RFI, and negatively associated with L-Glutamate, beta-Alanine, L-Tyrosine, and PC [18:2(9Z,12Z)/20:4(5Z,8Z,11Z,14Z)] in this study. In this study, the genus *Christensenellaceae_R-7_group*, *Lachnospiraceae_NK3A20_group*, and *Ruminococcus_2* were all associated negatively with RFI. Moreover, the genus *Christensenellaceae_R-7_group* was positively associated with L-Glutamate, beta-Alanine, L-Tyrosine, L-Aspartic Acid, L-Serine, phosphocholine, LysoPC [18:1(9Z)], LysoPC [18:1(11Z)], LysoPC [20:4(8Z,11Z,14Z,17Z)], LysoPC [20:4(5Z,8Z,11Z,14Z)], LysoPC [20:2(11Z,14Z)], PC [18:2(9Z,12Z)/20:4(5Z,8Z,11Z,14Z)]. The genus *Lachnospiraceae_NK3A20_group* was positively associated with L-Glutamate, L-Methionine, L-Isoleucine, L-Serine, L-Aspartic Acid, PC [18:2(9Z,12Z)/20:4(5Z,8Z,11Z,14Z)], PS [18:0/20:4(8Z,11Z,14Z,17Z)]. The genus *Ruminococcus_2* was positively associated with L-Glutamate, L-Aspartic Acid, L-Serine, beta-Alanine, choline, phosphocholine, PS [18:0/20:4(8Z,11Z,14Z,17Z)], LysoPC [18:1(9Z)], LysoPC [18:1(11Z)], LysoPC (16:0), LysoPC [20:4(8Z,11Z,14Z,17Z)], and LysoPC [20:4(5Z,8Z,11Z,14Z)]. Additionally, Brautigan et al. (2017) and Malanka et al. (2012) reported that LysoPCs affect nutrient absorption in the intestinal tract, and it also plays a regulator of immunological cell functions. Therefore, *Christensenellaceae_R-7_group*, *Lachnospiraceae_NK3A20_group*, and *Ruminococcus_2* might have a role in the metabolisms of amino acids metabolism and glycerophospholipid metabolism; and these bacterias increased antioxidant capacity and promoted nutrient digestion and absorption, which likely provides evidence supporting the higher feed efficiency in the LRFI group.

## Conclusion

In summary, the RFI phenotype significantly altered the relative abundances of certain intestinal bacteria communities, the genera *Christensenellaceae_R-7_group*, *Lachnospiraceae_NK3A20_group*, and *Ruminococcus_2* were negatively associated with RFI, while the genus *Acinetobacter* was positively associated with RFI. The RFI phenotype also significantly altered the concentrations of some intestinal metabolites, such as amino acids and glycerophospholipids. Additionally, the correlation between intestinal microorganisms and metabolites revealed that some microorganisms play an important role in amino acid metabolism, glycerophospholipid metabolism, nutrient digestion and absorption, and antioxidant enhancement, which likely provides evidence supporting the higher feed efficiency in the LRFI group. The predominant bacterial communities and the significantly differential metabolites in different SI segments were both common and unique, which suggested that microorganisms in different GIT locations might contribute separately to the RFI phenotype of beef cattle. Future, due to the individual variation of animals, samples from more animals should be analyzed to confirm these findings. Integrative information about the interactions between the SI microbial composition and metabolites in beef cattle with different RFIs could provide a better understanding of the small intestinal microbial and metabolites functions, allowing the development of improved strategies to increase feed efficiency. In addition, the mechanisms of the interactions among SI bacteria, metabolisms, and the RFI deserve further investigation.

## Data Availability Statement

The original contributions presented in the study are publicly available. This data can be found at: https://www.ncbi.nlm.nih.gov/, PRJNA796465.

## Ethics Statement

The animal study was reviewed and approved by the Laboratory Animal Welfare and Animal Experiment Ethical Committee of China Agricultural University (Protocol No. AW08059102-2).

## Author Contributions

QM, ZZ, and HW designed the research. YL and CL conducted the research. YL and CL analyzed the data. YL wrote the manuscript. QM and HW modified the manuscript. ZZ had responsibility for the final content. All authors read and approved the final manuscript.

## Funding

This work was supported by grants from the National Natural Science Foundation of China (grant number: 31972593), the Government Purchase Service (grant number: 16200158), and the China Agricultural Research System (grant number: CARS-37).

## Conflict of Interest

The authors declare that the research was conducted in the absence of any commercial or financial relationships that could be construed as a potential conflict of interest.

## Publisher’s Note

All claims expressed in this article are solely those of the authors and do not necessarily represent those of their affiliated organizations, or those of the publisher, the editors and the reviewers. Any product that may be evaluated in this article, or claim that may be made by its manufacturer, is not guaranteed or endorsed by the publisher.

## Footnotes

^1^ http://www.i-sanger.com/

^2^ http://www.hmdb.ca/

^3^ https://metlin.scripps.edu/

## Abbreviations

SI: Small intestine
HRFI: High residual feed intake
LRFI: Low residual feed intake
DMI: Dry matter intake
ADG: Average daily gain
MBW: Metabolic body weight
