# Supplementary material for: Small Intestine Microbiome and Metabolome of High and Low Residual Feed Intake Angus Heifers
Source: Front Microbiol. 2022 Apr 21;13:862151. doi: 10.3389/fmicb.2022.862151 (PMC9069012; doi:10.3389/fmicb.2022.862151)
Supplement: Supplementary file 1 [file Data_Sheet_1.docx]

Supplementary Material

# Supplementary Figures and Tables

## Supplementary Figures

Supplementary Figure 1 shows the overlap of the total ion chromatogram of the QC sample in the positive (A) and negative (B) ion modes, respectively. The results confirm the reliable repeatability and precision of the data obtained in the present study.

Supplementary Figure 2 shows samples examined by PCA following the positive (A) and the negative (B) mode ionization to provide a global overview of the differences among the metabolite data.

**Supplementary Figure 1**

**A**


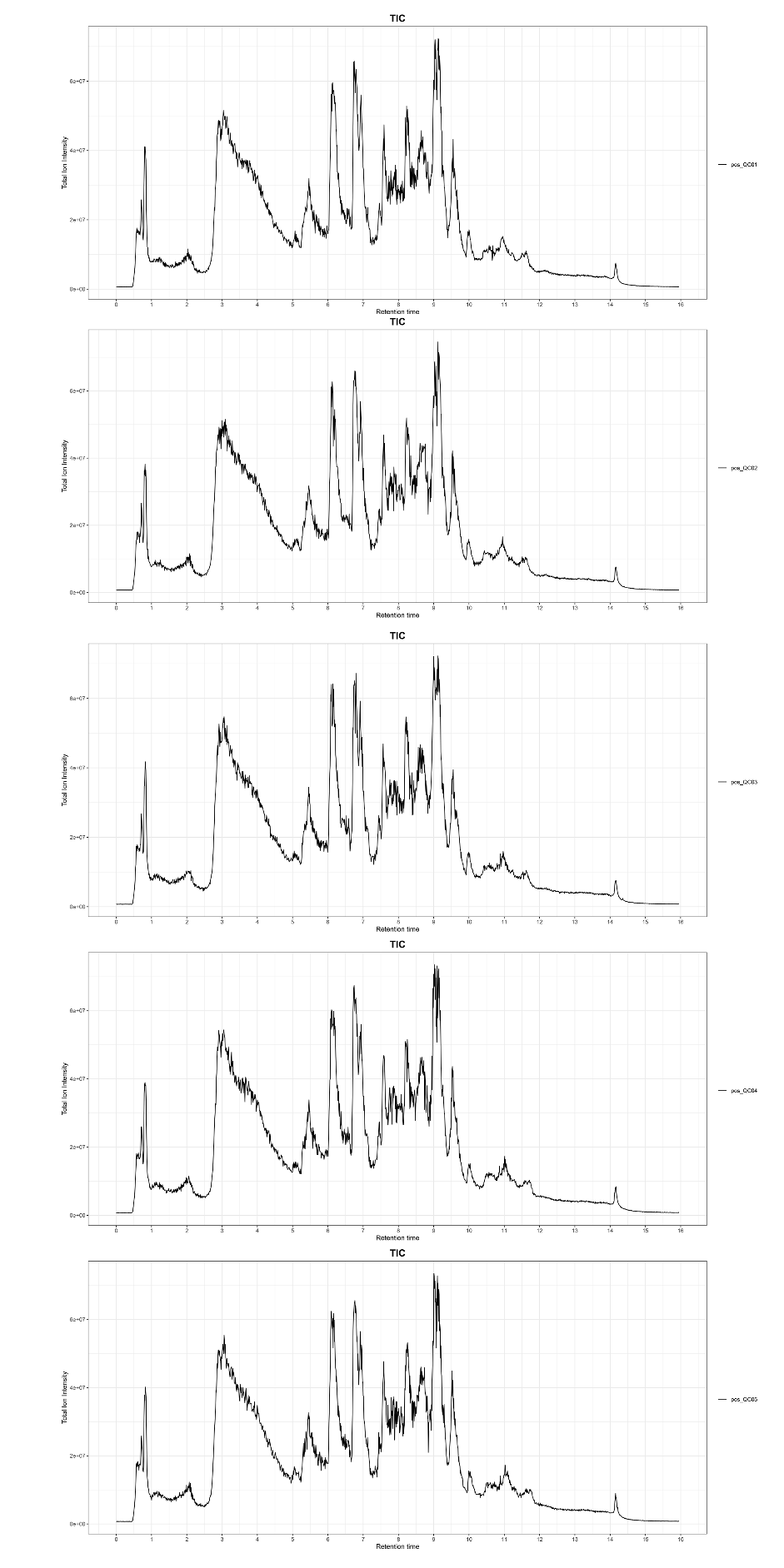


**B**


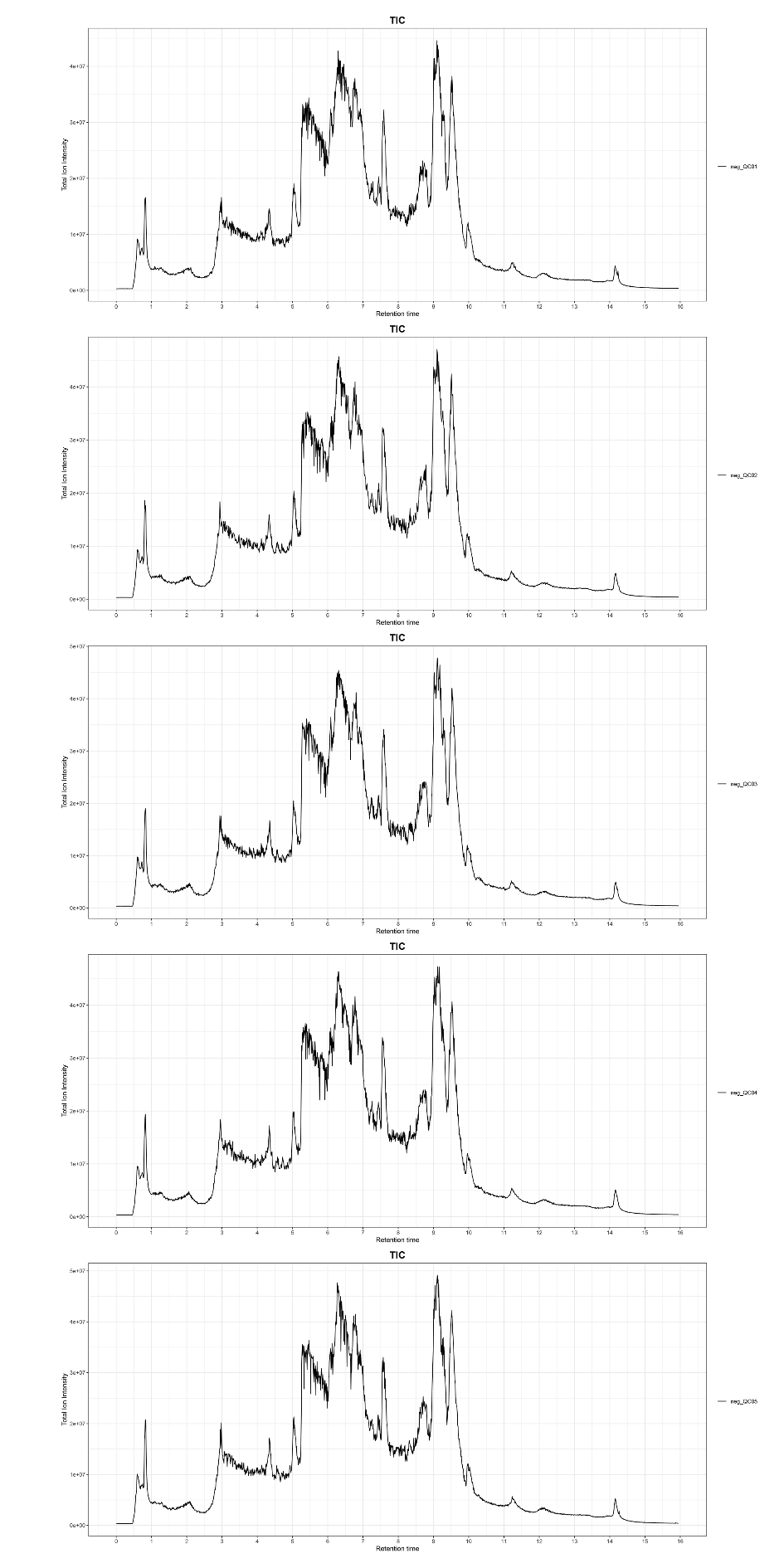


**Supplementary Figure 1.** LC-MS total ion chromatogram of the QC sample in the positive **(A)** and the negative **(B)** ion modes, respectively.

**Supplementary Figure 2**

**A**


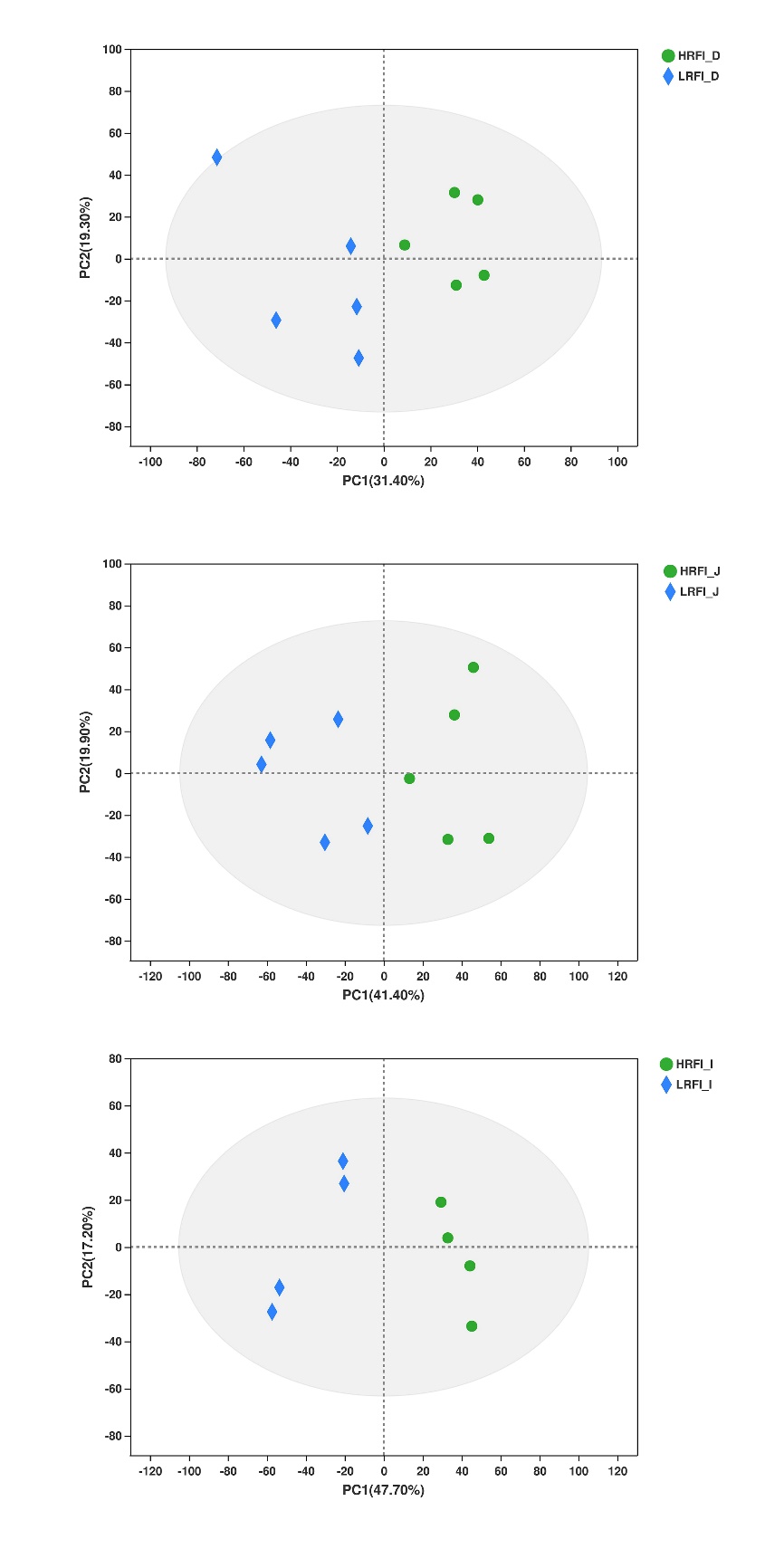


**B**

**
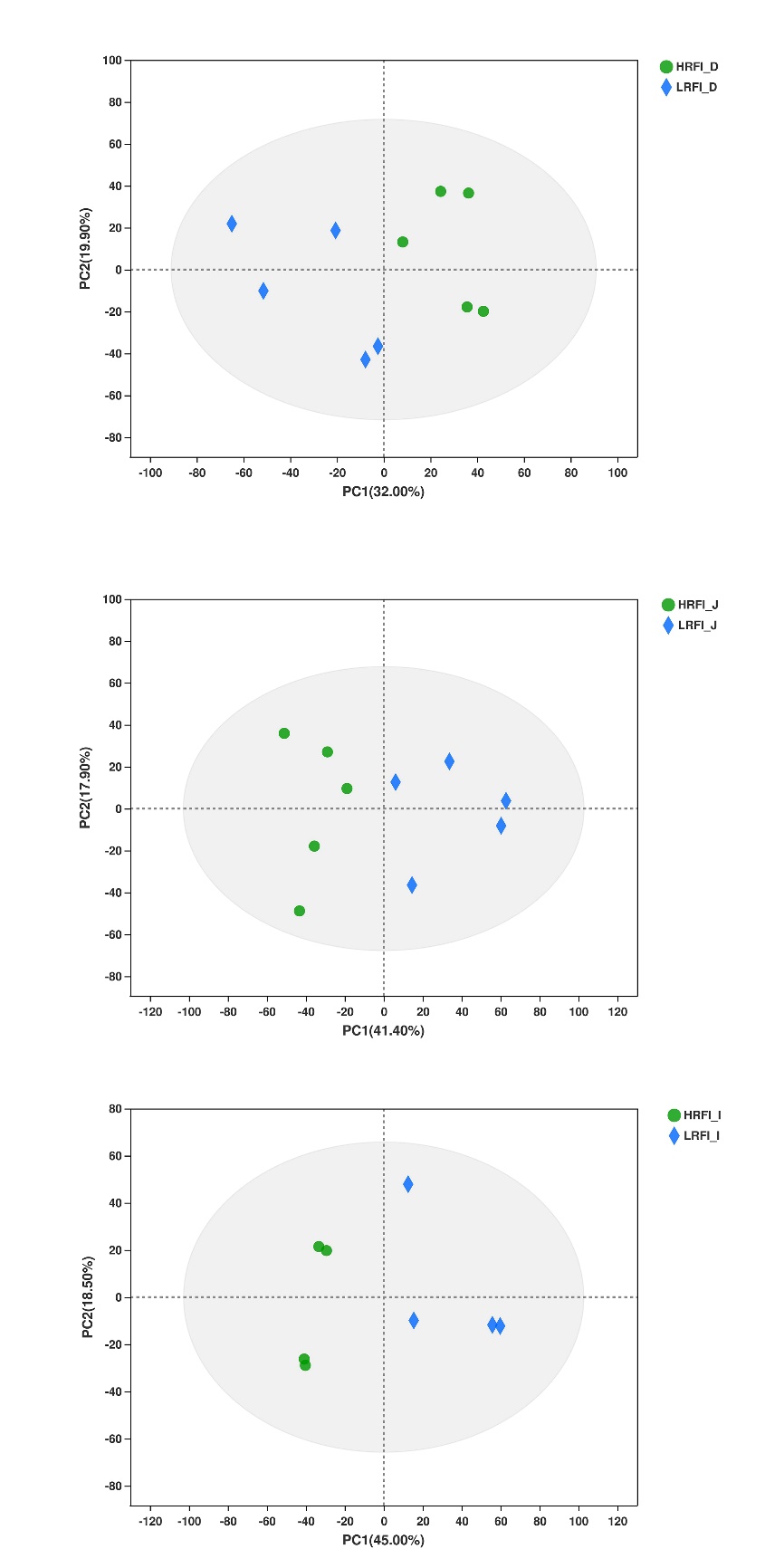
**

**Supplementary Figure 2.** The PCA plot of the ruminal samples corresponding to the HRFI and LRFI groups following the positive **(A)** and the negative **(B)** mode ionization, respectively.

## Supplementary Tables

**Supplementary Table 1.** Ingredients and chemical composition of the experimental diet.

| **Item** | **Value** |
| --- | --- |
| Ingredient composition, % DM |  |
| Whole corn silage | 30.00 |
| Wheat shell powder | 20.00 |
| Corn | 28.00 |
| Cottonseed meal | 10.50 |
| Corn germ meal | 10.00 |
| Premix^1^ | 0.20 |
| Salt | 0.50 |
| MgO | 0.20 |
| Limestone | 0.60 |
| Chemical composition |  |
| ME, Mcal/kg | 2.59 |
| NEg, Mcal/kg | 1.09 |
| TDN, % DM | 70.28 |
| NDF, %DM | 43.62 |
| CP | 13.25 |
| Ca | 0.38 |
| P | 0.32 |

^1^Mineral supplement contains 0.198% cobalt as well as 0.9228% copper, 8.0376% iron, 0.0754% iodine, 5.8131% manganese, 0.0366% selenium, and 6.635% zine.

**Supplementary Table 2.** Residual feed intake (RFI) of the 42 Angus heifers evaluated in this study.

| **Animal number** | **RFI** | **Group** ^1^ |
| --- | --- | --- |
| **30** | **-1.951** | **LRFI** |
| **51** | **-1.823** | **LRFI** |
| **17** | **-1.662** | **LRFI** |
| **40** | **-0.998** | **LRFI** |
| **49** | **-0.825** | **LRFI** |
| 66 | -0.703 | LRFI |
| 57 | -0.663 | LRFI |
| 3 | -0.654 | LRFI |
| 62 | -0.614 | LRFI |
| 1 | -0.436 | LRFI |
| 86 | -0.413 | LRFI |
| 15 | -0.387 | LRFI |
| 10 | -0.351 | NS |
| 11 | -0.329 | NS |
| 56 | -0.307 | NS |
| 44 | -0.181 | NS |
| 72 | -0.155 | NS |
| 5 | -0.133 | NS |
| 71 | -0.095 | NS |
| 80 | -0.028 | NS |
| 8 | 0.008 | NS |
| 73 | 0.053 | NS |
| 12 | 0.217 | NS |
| 50 | 0.332 | NS |
| 14 | 0.350 | NS |
| 48 | 0.398 | HRFI |
| 24 | 0.411 | HRFI |
| 37 | 0.441 | HRFI |
| 13 | 0.461 | HRFI |
| 82 | 0.520 | HRFI |
| 55 | 0.541 | HRFI |
| 68 | 0.545 | HRFI |
| 2 | 0.614 | HRFI |
| 7 | 0.664 | HRFI |
| 55 | 0.709 | HRFI |
| 37 | 0.719 | HRFI |
| 28 | 0.729 | HRFI |
| **29** | **0.757** | **HRFI** |
| **39** | **0.843** | **HRFI** |
| **45** | **0.904** | **HRFI** |
| **36** | **0.907** | **HRFI** |
| **64** | **1.584** | **HRFI** |

^1^ Bold line represent the 12 heifers selected for evaluation in this study; NS, -0.38 < RFI < 0.38.

**Supplementary Table 3.** Animal performances of the 10 Angus heifers selected in the present study.

| Animal number | Initial weight, kg | DMI, kg/d | ADG, kg/d | RFI, kg/d |
| --- | --- | --- | --- | --- |
| **LRFI** |  |  |  |  |
| 30 | 420.45 | 7.01 | 1.34 | -1.951 |
| 51 | 424.31 | 7.15 | 1.20 | -1.823 |
| 17 | 415.42 | 6.58 | 0.66 | -1.662 |
| 40 | 386.15 | 7.74 | 0.88 | -0.998 |
| 49 | 417.10 | 8.77 | 0.98 | -0.825 |
| **HRFI** |  |  |  |  |
| 29 | 434.62 | 8.80 | 1.00 | 0.757 |
| 39 | 429.50 | 10.12 | 0.86 | 0.843 |
| 45 | 399.68 | 10.38 | 0.93 | 0.904 |
| 36 | 433.32 | 9.05 | 0.74 | 0.907 |
| 64 | 425.15 | 9.91 | 0.66 | 1.584 |

LRFI: low residual feed intake; HRFI: high residual feed intake; DMI: dry matter intake; ADG: average daily gain.

**Supplementary Table 4.** Alpha diversity in duodenum, jejunum, and ileum bacterial communities between the HRFI and LRFI groups.

| **Small intestine** | **Items** | **HRFI** ^1^ | **LRFI** ^2^ | **SEM** ^3^ | ***P-*value** ^4^ |
| --- | --- | --- | --- | --- | --- |
| **Duodenum** |  |  |  |  |  |
|  | Chao 1 | 806.40 | 1208.60 | 274.06 | 0.06 |
|  | Shannon | 4.08 | 4.83 | 0.62 | 0.09 |
|  | Simpson | 0.08 | 0.05 | 0.04 | 0.21 |
| **Jejunum** |  |  |  |  |  |
|  | Chao 1 | 518.77 | 638.23 | 102.62 | 0.11 |
|  | Shannon | 2.72 | 3.54 | 1.00 | 0.25 |
|  | Simpson | 0.23 | 0.12 | 0.13 | 0.26 |
| **lleum** |  |  |  |  |  |
|  | Chao 1 | 561.76 | 572.31 | 50.84 | 0.78 |
|  | Shannon | 2.46 | 3.22 | 0.41 | 0.04 |
|  | Simpson | 0.24 | 0.14 | 0.07 | 0.09 |

^1^ HRFI, high residual feed intake. ^2^ LRFI, low residual feed intake. ^3^ SEM, standard error of the mean. ^4^ *P* values are derived using a Student’s t-test to assess the diferences between the HRFI and LRFI groups.
